# Age-Related Changes in Urethral Structure and Responds to Injury: Single-Cell Atlas of a Rat Model of Vaginal Birth Injury induced Stress Urinary Incontinence

**DOI:** 10.21203/rs.3.rs-3901406/v1

**Published:** 2024-02-12

**Authors:** Liangyu Zhao, Emily Xing, Tian Bai, Thomas Cao, Guifang Wang, Lia Banie, Guiting Lin, Yuxin Tang, Tom Lue

**Affiliations:** University of California, San Francisco; UCSF; UCSF; UCSF; UCSF; UCSF; UCSF; University of California San Francisco; University of California San Francisco

**Keywords:** Stress urinary incontinence (SUI), vaginal balloon dilation (VBD), single cells RNA sequence, Senescence-Associated Secretory Phenotype (SASP), urethra

## Abstract

Stress urinary incontinence (SUI) greatly affects the daily life of numerous women and is closely related to a history of vaginal delivery and aging. We used vaginal balloon dilation to simulate vaginal birth injury in young and middle-aged rats to produce a SUI animal model, and found that young rats restored urethral structure and function well, but not the middle-aged rats. To identify the characteristics of cellular and molecular changes in the urethral microenvironment during the repair process of SUI. We profiled 51,690 individual female rat urethra cells from 24 and 48 weeks old, with or without simulated vaginal birth injury. Cell interaction analysis showed that signal networks during repair process changed from resting to active, and aging altered the distribution but not the overall level of cell interaction in the repair process. Similarity analysis showed that muscle, fibroblasts, and immune cells underwent large transcriptional changes during aging and repair. In middle-aged rat**s**, cell senescence occurs mainly in the superficial and middle urothelium due to cellular death and shedding, and the basal urothelium expressed many Senescence-Associated Secretory Phenotype (SASP) genes. In conclusion, we established the aging and vaginal balloon dilation (VBD) model of female urethral cell anatomy and the signal network landscape, which provides an insight into the normal or disordered urethra repair process and the scientific basis for developing novel SUI therapies.

## INTRODUCTION

The International Continence Society defines stress urinary incontinence (SUI) as involuntary loss of urine with effort or physical exertion^1^. Vaginal delivery and aging are two important risk factors for SUI, and the two risk factors also synergistically exacerbate SUI symptoms^2,3^. In a previous study, we simulated vaginal birth injury by prolonged vaginal balloon dilation (VBD) and oophorectomy, many rats recovered from induced tissue injury and regain continence spontaneously after 4–6 weeks^4^. To produce a long-term SUI model, we added lysyl oxidase (LOX) inhibitor, b-aminopropionitrile (BAPN), to disrupt the crosslinking of collagen and elastin, and created an irreversible SUI model^5^.

Previously, the mainstream view is that SUI is related to the inability of the pelvic floor muscles to maintain bladder neck position during straining. Therefore, pelvic floor muscle exercise–based behavioral therapy is the foundation of nonpharmacologic management^6,7^. Recently studies have highlighted the importance of urethral closing pressure in maintaining urinary continence^8,9^. Pelvic floor and urethral striated muscle were regarded as the most important functional cell type in producing and maintaining urethral pressure. In addition, weakened sealing effect of the urethral mucosa, decreased elasticity of the urethral wall and shortened length of the functional urethra together also led to the occurrence of urinary incontinence^10^.

Since urethral tissue from young, old and SUI women are difficult to obtain, we performed a single-cell RNA sequencing (scRNA) technology in our established SUI rat models to explore the transcriptional changes of various cells and the cell-cell interactions in the tissue microenvironment at the single-cell and molecular scales^11–13^. Henry et al, reported the first cellular anatomy of the normal adult human prostatic urethra, and created a powerful resource for experimental design in human prostate disease^14^. Nevertheless, there exists significant differences of physiological basis and pathological anatomy between male and female SUI. To improve our understanding of the pathophysiology of SUI, we profiled 51,690 individual female rat urethra cells from 24 and 48 weeks old, with or without VBD or BPAN treatment. Based on these data, we developed a single-cell atlas of the female urethra and described the communication network within its microenvironment in normal and injured urethral tissues.

## RESULTS

### Overview of the cellular composition of the rat urethra

**Figure 1:**
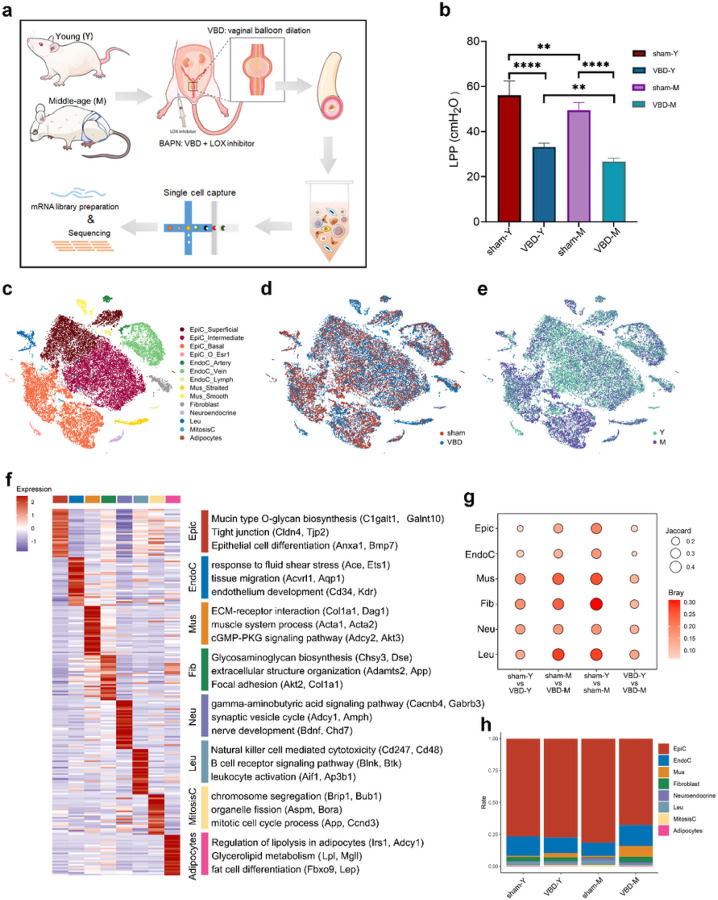
Global expression profiling of normal and urinary incontinence rat urethra cells by single-cell RNA-seq. (a) Schematic illustration of the experimental workflow in this study. (b) Urodynamic functional study. Leak point pressure (LPP). (c-e) t-distributed stochastic neighbour embedding (tSNE) plots of all urethra cells from normal and urinary incontinence rat. Cells are coloured according to their (c) cell types, (d) treatment groups, or (e) age. (f) Heatmap of the top 30 DEGs in each major cluster (left panel), with the GO analysis (biological process) according to the DEGs of each major cluster (right panel). The top2 specific marker genes are labelled in parentheses. A gradient of light blue to dark red indicates low to high expression levels in the heatmap. (g) Dissimilarity of 6 major urethra cell clusters between groups are shown on bubble diagram. The gradient of bubbles sizes indicates low to high scaled Jaccard distance, and the gradient of red indicates low to high scaled Bary values. (h) Bar plot showing the cell count proportion of 8 major cell clusters in each group. VBD: vaginal balloon dilation; BAPN: b-aminopropionitrile + VBD; Epic: epithelial cell; EndoC: endothelial cells; Mus: muscle cells; Fib: fibroblast; Neu: neuroendocrine cells; Leu: leukocytes.

To characterize the rat urethral cellular heterogeneity and cellular diversity related to aging and injuries, we profiled urethral cells from young (Y, 24 weeks) and middle-aged (M, 48 weeks) rats with or without vaginal balloon dilation (VBD group), and VBD combined with LOX inhibitor injection (BAPN group) (Fig. 1a). We found both age and VBD independently and significantly decreased the leak point pressure (LPP) of rat urethra (Fig. 1b).

A total of 51,690 cells were retained after removing empty droplets, outliers, cell debris, and inferential doublets. Eight major clusters were identified in the whole cell population based on the expression of known cell type-specific markers, including epithelial cells (EpiC), endothelial cells (EndoC), muscle cells (Mus), fibroblast (FBs), neuroendocrine, leukocyte (Leu), mitotic cells (MitosisC), and adipocytes (Fig. 1c and Supplementary Fig. 1a). These 8 clusters could be further divided into 14 subclusters (Fig. 1c). In the sham and VBD group, young and middle-age rat cells showed significant heterogeneity in t-SNE plot (Fig. 1d and 1e). Middle-age rat cells showed higher transcription levels than young rat (Supplementary Fig. 1b). Each subcluster also had different gene transcription levels (Supplementary Fig. 1c and 1d). A total of 7,190 differentially expressed genes (DEGs) among the 8 clusters were identified (Fig. 1f and Supplementary Table 1). Gene ontology (GO) analysis was performed for the top 30 DEGs of each cluster, the results are consistent with the previous understanding of the biological characteristics of these cell types (Fig. 1f). To identify the cell composition that is highly variable in the aging and incontinence models, we compared each group in each cell cluster and found that Mus, Fib, and Leu clusters showed bigger transcriptional changes than other cells (Fig. 1g). EpiC and EndoC accounted for the majority of the urethral microenvironment, and the proportion of each cell cluster was not significantly different in each group (Fig. 1h). To explore whether age-related changes have occurred in the transcription patterns of urethral tissue in middle-aged rats, we also invoked two age-related datasets^15,16^, which showed that the expression of these genes in middle-aged rats were significantly higher than that in young rats (Supplementary Fig. 2a and 2b).

### Characteristics of urethral regeneration in young rats

**Figure 2:**
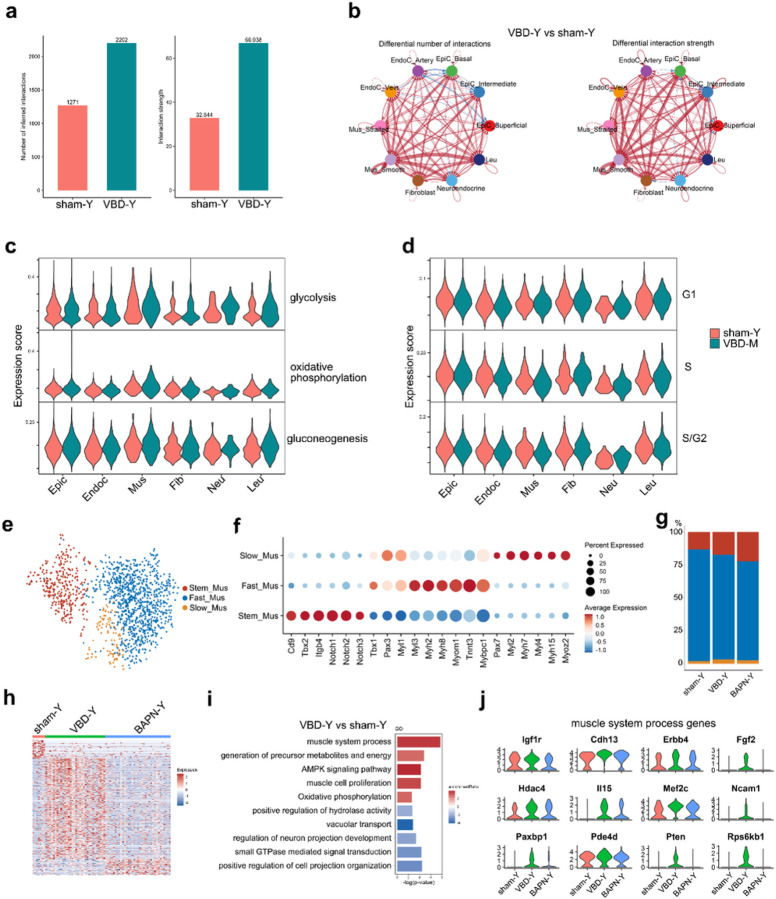
Transcriptional changes of cell microenvironment during urethral injury repair. (a) The barplot showing the total interaction count and strength within urethral microenvironment of young normal and VBD rat. (b) Circle plots showing the up- or down-regulation of cell–cell communication network between young normal and VBD rat. The red line represents up-regulated interaction and the blue line represents down-regulated. (c-d) Violin plot showing the expression level of the genes that are associated with (c) energy metabolism and (d) cell cycle. (e) Uniform manifold approximation and projection (UMAP) dimensionality reduction plot of all rat striated muscle of urethra. Cells are coloured according to their types. (f) Bubble diagram showing the marker genes of each striated muscle cluster. The gradient of bubbles sizes indicates low to high expressed percent, and the gradient of blue to red indicates low to high average expression level. (g) Bar plot showing the cell count proportion of 3 striated muscle clusters in each group. (h) Heatmap of the DEGs in 3 striated muscle clusters. A gradient of light blue to dark red indicates low to high expression levels in the heatmap. (i) Bar plot showing the GSEA (biological process and KEGG pathways) terms according to the DEGs between sham24 and VBD24 striated muscles. Statistical analysis was based on Fisher's exact test; two-tailed; the confidence interval is 95%. A gradient of light blue to red indicates negative to positive enrichment of the term. The length of the bar indicates the *P* value. (j) Violin plot showing the expression level of genes belong to “muscle system process” terms among sham24, VBD24 and BAPN24 striated muscles. VBD: vaginal balloon dilation; BAPN: b-aminopropionitrile + VBD; Epic: epithelial cell; EndoC: endothelial cells; Mus: muscle cells; Fib: fibroblast; Neu: neuroendocrine cells; Leu: leukocytes.

Urethral injury repair is a complex process subject to strict and precise signal regulation. We found that the number and intensity of cell-cell signal pairs in the VBD group were much greater than those in the sham group (Fig. 2a). Almost all interactions between cell clusters were upregulated in VBD, suggesting that urethral repair requires mobilization of a large number of signal activation in the microenvironment (Fig. 2b). Specific to each signalling, the VBD group and the sham group showed greater differences in “COLLAGEN”, “Cadherin (CDH)”, “VEGF”, et al (Supplementary Fig. 3a, 3b and 3c). In terms of energy metabolism, we also found that the expression of genes related to glycolysis and oxidative phosphorylation in Mus, Neu and Leu cluster in the VBD group was higher than that in the control group (Fig. 2c). In addition, although there were slightly more mitotic cells in the VBD group than in sham group, there were no differences in the expression of cell cycle-related genes in other cell types between the two groups (Fig. 2d).

According to the above cellular differences and our previous studies, muscle cells, especially urethral striated muscle, are crucial for the generation of urethral pressure, and the recovery of its function is closely related to the improvement of clinical symptoms of urinary incontinence. We found that smooth muscle and striated muscle can be distinguished by expression patterns of genes such as *Ttn*, *Neb*, *Mybpc1*, *Mlip*, *Myh11*, *Mylk*, *Actg2* and *Acta2* (Supplementary Fig. 4a and 4b). Next, we found that the striated muscle can be further divided into stem cell subcluster (Stem_Mus), fast muscle subcluster (Fast_Mus) and slow muscle subcluster (Fast_Mus), and the three clusters have unique gene expression patterns of muscle phylogeny (Fig. 2e and 2f). In terms of the number of cells, the proportion of Stem_Mus increased successively in sham, VBD and BAPN groups. However, VBD24 stem cells had more overexpressed genes than sham and BAPN group (Fig. 2g). According to functional enrichment analysis of differential genes in stem cells of VBD and sham group (Fig. 2h), we found significant positive enrichment of “muscle system process”, “generation of precursor metabolites and energy” and “muscle cell proliferation” (Fig. 2i). We were surprised to find that *Esr1* was only expressed in smooth muscle and was significantly elevated in the VBD and BAPN groups (Supplementary Fig. 4c, 4d and 4e). Smooth muscle maintains urethral pressure in coordination with striated muscle. Based on the differential smooth muscle genes of VBD and control group, we found that “response to estradiol” was significantly enriched, while related genes were successively increased in sham, VBD and BAPN groups, suggesting the role of estrogen in smooth muscle repair (Supplementary Fig. 4f and 4g). Genes related to muscle system process such as *Igf1r*, *Cdh13* and *Fgf2* were significantly highly expressed in VBD Stem_Mus (Fig. 1j). In the cell interactions associated with striated muscle, “LAMININ” and “AGRN” signaling was significantly upregulated in VBD, suggesting the importance of extracellular matrix and nerve regulation (Supplementary Fig. 5a and 5b).

### Aging alters the microenvironmental signaling network during urethral repair

**Figure 3:**
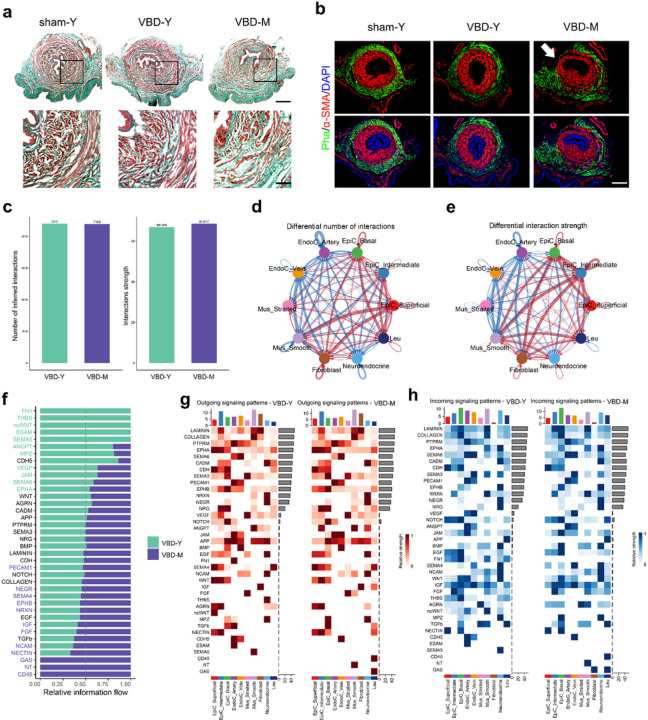
Cell–cell communication network within the urethra microenvironment of young and old VBD rat. (b) The barplot showing the total interaction count and strength within urethral microenvironment of young and old VBD rat. (c-d) Circle plots showing the total (c) interaction count and (d) strength of cell–cell communication network between young and old VBD rat. The red line represents up-regulated interaction and the blue line represents down-regulated. (e) The barplot showing the relative information flow between young and old VBD rat urethra microenvironment. Green or purple fonts represent signals with statistical differences. (f) Heatmap of the outcoming signals of each cluster between young and old VBD rat urethra microenvironment. A gradient of white to dark red indicates low to high expression weight values in the heatmap. (g) Heatmap of the outcoming signals of each cluster between young and old VBD rat urethra microenvironment. A gradient of white to dark blue indicates low to high expression weight values in the heatmap.

In our previous studies in rats underwent VBD, only the young rat group recovered near normal leak point pressure^17–19^. In Masson trichrome staining we noted a decrease in both urethral smooth and striated muscles and an increase in extracellular matrix in middle-aged VBD groups (Fig. 3a). Interruption of circular striated muscle layer (Pha staining positive layer) was also noted in some rats of middle-aged VBD group (Fig. 3b). Surprisingly, there was little difference in the number and strength of cell-cell interactions in the urethral microenvironment between the young and middle-aged VBD rats (Fig. 3c). However, the interaction of arterial endothelium, venous endothelium, smooth muscle, and striated muscle with other cells decreased, while cellular communication between several other cell subclusters increased (Fig. 3d and 3e). It is suggested that the dysfunction of urinary tract repair in older rats is related to the pattern of signal distribution rather than the change of overall signal strength. In middle-aged VBD rats, FN1 and THBS signals were down-regulated, while CD45 and NT signal families were up-regulated (Fig. 3f). Specific to muscle cells, we found that signals involved in muscle regeneration and fibrosis, such as and TGFβ were significantly increased, while Wnt was significantly decreased, further suggesting the complexity of the effects of aging on urethral repair (Fig. 3g, 3h and Supplementary Fig. 6a).

### Overall transcriptional characteristics of aging in the urethral microenvironment of rats

**Figure 4:**
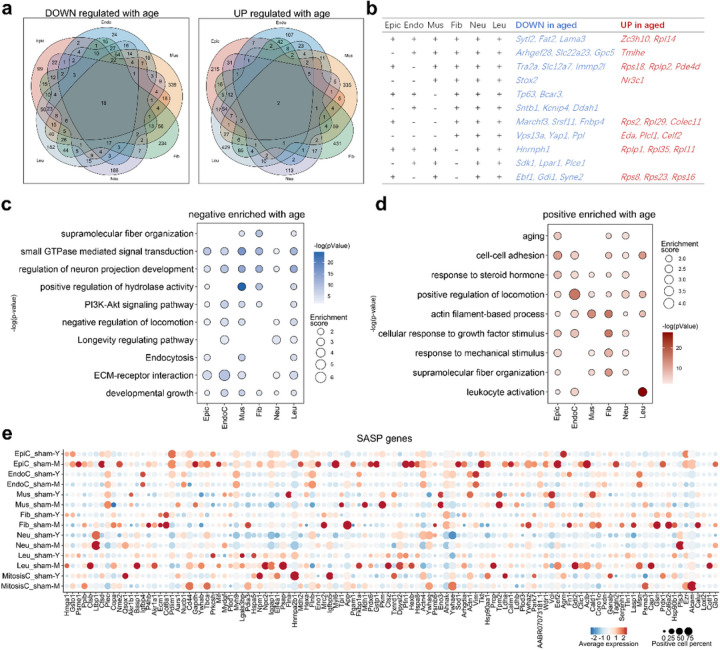
Overview of aging characteristics of urethra microenvironment. (a) The Venn diagram shows the up-regulated or down-regulated genes with age that are common or unique to each cell cluster. (b) The table showing the up-regulated or down-regulated genes with age that are common to some or all clusters. (c-d) Bubble diagram showing the GO analysis terms according to the (c) up-regulated DEGs or (d) down-regulated DEGs with age in each cluster. Statistical analysis was based on Fisher's exact test; two-tailed; the confidence interval is 95%. The gradient of bubbles sizes indicates low to high enrichment score, and the gradient of colour ranges from light to dark indicates high to low *P*value. (e) Bubble diagram showing the SASP genes of each cluster between young and old rat. The gradient of bubbles sizes indicates low to high expressed percent, and the gradient of blue to red indicates low to high average expression level.

Understanding the pattern of senescence of each cell type in the urethral microenvironment of rats at different age may help explain the less robust urethral repair mechanism in middle-aged rats. We compared different subtypes of cells in young and middle-aged rats, intersecting the six major cell subclusters, and obtained 18 down-regulated genes (include *Sytl2*, *Fat2*, *Lama2*, et al) and 2 up-regulated genes (*Zc3h10* and *Rpl14*) related to aging (Fig. 4a and 4b). Based on the DEGs of each subcluster between young and middle-aged, we noted that all type of cells has some common aging characteristics. For example, “small GTPase mediated signal transduction” and “regulation of neuron projection development” were negatively enriched, while “positive regulation of locomotion” and “actin filament-based process” were positively enriched in all cell types (Fig. 4c and 4d). In addition, ECM remolding-related terms such as “supramolecular fiber organization”, “positive regulation of hydrolase activity” and “response to mechanical stimulus” were also enriched in some clusters (Fig. 4c and 4d). SASP has been described as characteristic of senescence in multiple cell types^16^, but we found that the SASP gene was not universal in urethral tissue, and only small part of upregulated SASP genes were found in EpiC, Fib, and Leu clusters, suggesting the particularity of urethral senescence (Fig. 4e).

### Characteristics and signaling pathways of urethral muscle aging in rats

**Figure 5:**
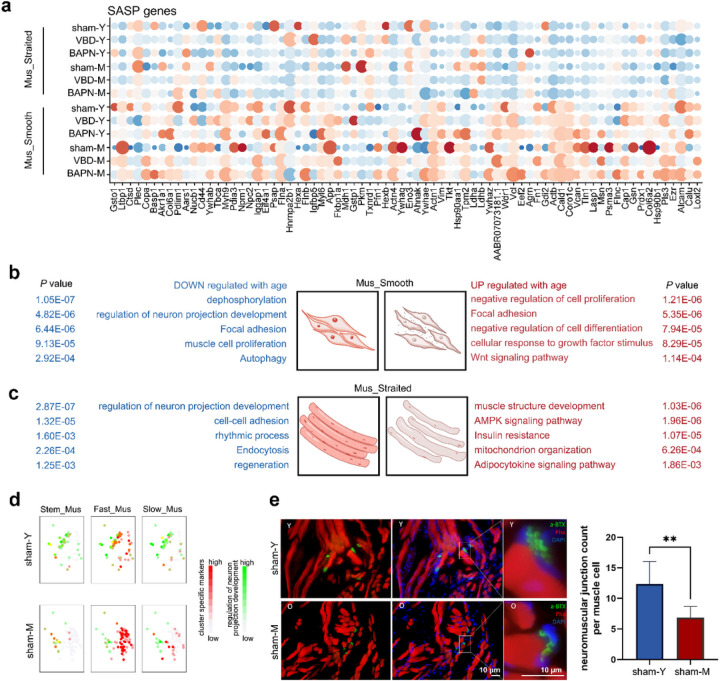
The aging characteristics of urethra muscle clusters. (a) Bubble diagram showing the SASP genes of each muscle cluster among young and old rat with or without VBD treatment. The gradient of bubbles sizes indicates low to high expressed percent, and the gradient of blue to red indicates low to high average expression level. (b-c) The GO analysis terms according to the up-regulated DEGs or down-regulated DEGs with age in (b) smooth muscle or (c) striated muscle cell cluster. Statistical analysis was based on Fisher's exact test; two-tailed; the confidence interval is 95%. (d) The UMAP plots showing the transcription pattern of genes belong to “regulation of neuron projection development” term (green) and striated muscle subtype characteristic genes (red). (e) Immunohistochemical co-staining of α-BTX (green) and Pha (red) in normal young and old urethra tissue paraffin sections. The right panel showing the statistics of neuron muscle junction count per muscle cell. The scale bar represents 10 µm.

Urethral pressure is mainly generated by muscle contraction, and its structural and functional disorders are closely related to urinary incontinence. Therefore, we focused on the aging changes of muscle cells, and found that smooth muscle and striated muscle exhibited different characteristics during aging. First, we compared the differences in the expression of classical SASP genes in striated muscle and smooth muscle of each group, and found that these genes showed a tendency of aging up-regulation only in smooth muscle (Fig. 5a). When using aging relate DEGs, we found “Wnt signaling pathway” term was up-regulated in the smooth muscle aging process. Interestingly, both up-DEGs and down-DEGs of smooth muscle aging were enriched to “Focal adhesion” (Fig. 5b). By focusing on this pathway, we found that the expression of integrin family, such as Itga1, Itgb1, Itgb6 and Igta9 was down-regulated during aging, but the expression of extracellular matrix related genes such as *Col1a1*, *Col2a2*, *Lama4* and *Lamb2*, et al were up-regulated (Supplementary Fig. 7a and 7b). In addition, the down-regulation of cell proliferation and autophagy may also be involved in the aging process of smooth muscle (Fig. 5b). Another interesting finding was that both striated muscle and smooth muscle were negatively enriched to “regulation of neuron projection development” term (Fig. 5b and 5c). Specific to each subcluster of striated muscle, we found that neuron projection development related genes showed more co-expression with characteristic genes of Fast_Mus, but decreased in middle-age rats (Fig. 5d). Immunohistochemical co-staining showed that the number of synapses at each neuromuscular junction of striated muscle cell in middle-aged rats was significantly lower than that of young rats (Fig. 5e). It is suggested that the changes of muscle structure and function are also related to the decline of nerve regulation.

### The aging roadmap of rat urethral fibroblast

**Figure 6.**
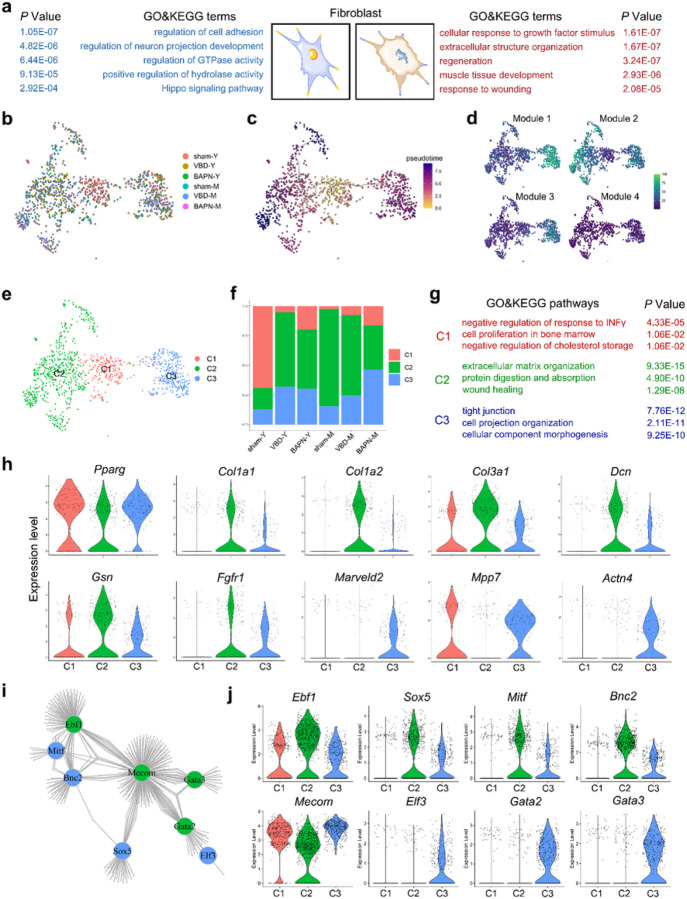
The aging characteristics of urethra fibroblast. (a) The GO analysis terms according to the up-regulated DEGs or down-regulated DEGs with age in urethra fibroblast. Statistical analysis was based on Fisher's exact test; two-tailed; the confidence interval is 95%. (b-e) The UMAP dimensionality reduction plot of rat urethra fibroblast cluster. Cells are coloured according to their (b) sample group, (c) pseudotime, (d) expression level of 4 models and (e) subclusters. (f) Bar plot showing the cell count proportion of 3 fibroblast clusters in each group. (g) The GO and KEGG analysis terms according to the up-regulated DEGs in each fibroblast subcluster. Statistical analysis was based on Fisher's exact test; two-tailed; the confidence interval is 95%. (h) Violin plot showing the expression level of genes belong to GO term “negative regulation of cholesterol storage” (*Pparg*), “wound healing” (*Col1a1*, *Col1a2*, *Col3a1*, *Dcn*, *Gsn*, *Fgfr1*) and “tight junction assembly” (*Marveld2*, *Mpp7*, *Actn4*) among 3 fibroblast subclusters. (i) The link plot showing the main regulators in C2 (green) and C3 (blue) subcluster. (j) Violin plot showing the expression level of main regulators among 3 fibroblast subclusters.

Fibroblasts are distributed throughout the stroma of the urethra and are essential for the formation of ECM. We found smooth muscle and fibroblasts are two main sources of collagens (Supplementary Fig. 8a). By comparing the urethral fibroblasts of young and middle-aged rats, we found that “cellular response to growth factor stimulus”, “extracellular structure organization” and “response to wounding” terms were significantly positively enriched with aging, suggesting the activation of fibroblasts (Fig. 6a). In addition, “regulation of cell adhesion”, “Hippo signaling pathway” are down-regulated, suggesting that the unbalance of cells and extracellular matrix interaction (Fig. 6a). To explore the activation lineage of fibroblasts, we performed uniform manifold approximation and projection (UMAP) dimensionality reduction and pseudo-time analysis, which showed that fibroblast activation had four distinct gene expression patterns and two distinct differentiation pathways (Fig. 6b, 6c and 6d). We identified the inactive fibroblasts as C1 subcluster, and the two activated fibroblast were named as C2 and C3, respectively (Fig. 6e). Different from the young sham group in which C1 fibroblast accounted for the majority, the C2 and C3 subclusters increased in the remaining groups (Fig. 6f). “negative regulation of response to INFγ”, “cell proliferation in bone marrow” and “negative regulation of cholesterol storage” GO terms were significantly enriched in C1 fibroblast, in addition, Pparg was highly expressed in this group, suggesting its characteristics of proliferation, anti-inflammatory and lipid metabolism regulation (Fig. 6g). “extracelluar matrix organization”, “protein digestion and absorption” and “wound healing” terms were enriched in C2 subclusters, suggesting C2 is ECM producing fibroblast and may be involved in the repair of urethral injury, but its increased proportion in aging and high expression of ECM genes suggest that it also may be related to urethral interstitial fibrosis (Fig. 6g, 6h and Supplementary Fig. 8b and 8c). C3 fibroblast is enriched to “tight junction”, “cell projection organization” and “cellular component morphogenesis” terms and has high expression of *Marveld2*, *Actn4*, and metalloproteinases suggests that it may play a role in barrier structures and ECM remodeling (Fig. 6g ,6h and Supplementary Fig. 8d). We also examined the main transcription factors that may regulate fibroblasts activation, and found that *Ebf1*, *Sox5*, *Mitf* and *Bnc2* may induce a C2 lineage, and *Mecom*, *Elf3*, *Gata2* and *Gata3* may play an important role in the C3 lineage (Fig. 6i and 6j).

### Changes of immune microenvironment in urethra of middle-age rats

**Figure 7:**
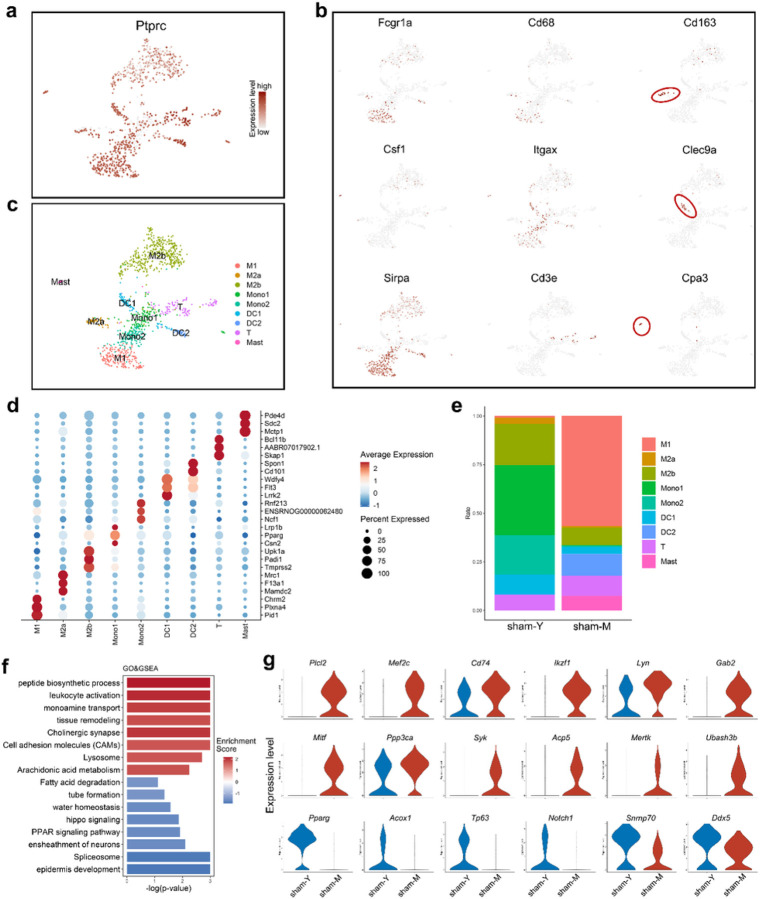
The aging state of urethra immune microenvironment. (a-c) The UMAP dimensionality reduction plot of rat urethra fibroblast cluster. Cells are coloured according to their (a) expression level of *Ptprc*, (b) expression level of each immune cell markers, and (c) subclusters. (d) Bubble diagram showing the top DEGs of each immune cell cluster. The gradient of bubbles sizes indicates low to high expressed percent, and the gradient of blue to red indicates low to high average expression level. (e) Bar plot showing the cell count proportion of 9 immune subclusters in young and old rat urethra. (f) Bar plot showing the GSEA (biological process and KEGG pathways) terms according to the DEGs between sham24 and VBD24 immune cells. Statistical analysis was based on Fisher's exact test; two-tailed; the confidence interval is 95%. A gradient of light blue to red indicates negative to positive enrichment of the term. The length of the bar indicates the *P*value. (g) Violin plot showing the expression level of genes belong to GO term “leukocyte activation” (upper and middle panel) and “PPAR signaling pathway” (bottom panel) between sham24 and VBD24 immune cells.

Aging is closely related to chronic inflammation, and homeostatic imbalance in the immune microenvironment which have been found in a variety of tissues and organs. In the urinary tract, we used *Cd45* (*Ptprc*) expression to screen out immune cell cluster, and then re-performed clustering and dimensionality reduction analysis (Fig. 7a). Referring to the classical immune cell-specific gene expression pattern, and finally obtained 9 immune subcluster (Fig. 7b and 7c). In addition, we further identified the highly expressed genes and potential biomarkers in these nine subpopulations (Fig. 7d). By comparing the relative proportion of these 9 subclusters in young and middle-aged rats’ urethral tissues, we found that M1 macrophages and mast cells increased significantly with aging (Fig. 7e). At the functional level, we found that the GO and KEGG terms such as “peptide biosynthetic process”, “leukocyte activation” and “tissue remodeling” were positively enriched with aging, while “epidermis development”, “PPAR signaling pathway” and “hippo signaling” were negatively enriched (Fig. 7f). The expression of genes belonging to “leukocyte activation” term such as *Plcl2*, *Mef2c*, *Cd47*, et al increased significantly in middle-aged rats, while PPAR-related genes such as *Acox1*, *Tp63* and *Notch1* decreased (Fig. 7g). These results suggest that the immune microenvironment may also play an important role in aging and repair process of the rat urethra.

### The classic aging phenotype occurs primarily in the Urothelium

**Figure 8:**
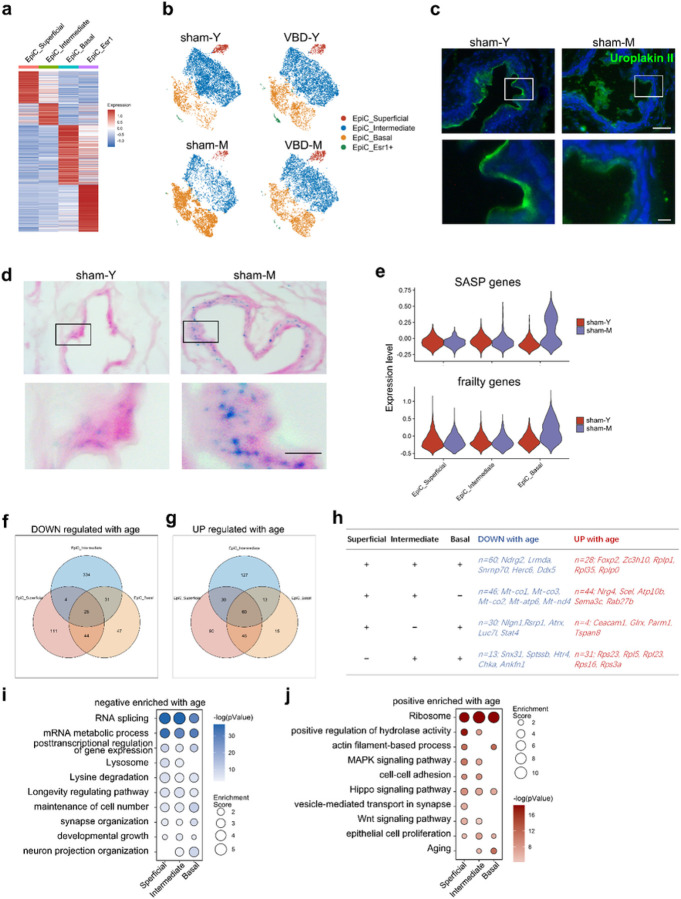
The heterogeneity of urothelium aging process. (a) Heatmap of the DEGs in 4 urothelium subclusters. A gradient of light blue to dark red indicates low to high expression levels in the heatmap. (b) The UMAP dimensionality reduction plot of rat urothelium cells. Cells are coloured according to their subcluster and plot is split by their groups. (c) Immunohistochemical staining of uroplakin (green) in normal young and old urethra tissue paraffin sections. (d) β-gal staining of normal young and old urethra tissue paraffin sections. The lower panel is the magnification of the upper field. The scale bar represents 50 µm (upper panel) and 10 µm (lower panel). (e) Violin plot showing the expression score of SASP and frailty genes between sham24 and VBD24 in different urothelium subclusters. (f-g) The Venn diagram shows the (f) up-regulated or (g) down-regulated genes with age that are common or unique to each urothelium subcluster. (h) The table showing the up-regulated or down-regulated genes with age that are common to some or urothelium subclusters. (i-j) Bubble diagram showing the GO analysis terms according to the (i) up-regulated DEGs or (j) down-regulated DEGs with age in urothelium subcluster. Statistical analysis was based on Fisher's exact test; two-tailed; the confidence interval is 95%. The gradient of bubbles sizes indicates low to high enrichment score, and the gradient of colour ranges from light to dark indicates high to low *P*value.

In our data, epithelial cells accounted for the largest proportion of cell type in the urethral microenvironment (Fig. 1c). However, epithelial cells of young rats did not show significant changes 2 weeks after VBD, suggesting that the urothelium had been completely repaired (Fig. 1g). Interestingly, we found that the urothelium of middle-aged rats showed large differences between the VBD and sham groups (Fig. 1g). We speculate it may be related to the age-related decline in repair capacity of urothelium. We further divided the urothelium into four subclusters based on gene expression patterns (Fig. 8a and Supplementary Fig. 9a), and found that superficial and intermediate epithelial cells decreased in middle-aged rats, while basal epithelial cells increased (Fig. 8b). We then confirmed this result using immunofluorescence staining, showing decreased expression of UPK3 in the urothelium of middle-aged rats and partial exfoliation of superficial UPK3 positive cells (Fig. 8c). Next, we found that β-gal positive cells were distributed throughout the whole layer of the urothelium in middle-aged rats. However, in contrast to the basal epithelial cells, the SASP and frailty genes expressed by superficial and intermediate epithelial cells of middle-aged rats were not significantly different from those of young rats, suggesting that basal cells may be the initiator of aging of the entire urothelial microenvironment (Fig. 8e, Supplementary Fig. 10a and 10b). Total 60 genes were down-regulated and 28 genes were up-regulated with aging in all three types of urothelial cells (Fig. 8f, 8g and 8h). In middle-aged rats, GO and KEGG terms such as “RNA splicing” and “mRNA metabolic process” were negatively enriched in all three cell clusters, corresponding to a positive enrichment for “Ribosome”, suggesting the importance of post-transcriptional and translational regulation in the aging process of urothelium (Fig. 8i and 8j).

## DISCUSSION

Urinary incontinence is referred as a “social cancer”, which greatly affect women's quality of life. Among women aged 20 years or older, 17.1% of them have moderate to severe UI, and this proportion is as high as 30–40% in older age^1,20^. The worldwide incidence of SUI was projected to be 167 million people in 2019 ^21^. In this study, we used rats of age 24 and 48 weeks to represent young and middle-aged women. We also used VBD with or without BAPN to create long term or short-term SUI models respectively^4,5^. Single-cell sequencing technology was used to explore the transcriptional changes of each cell in the whole urethral microenvironment, as well as the signal communication changes among cells. This study helps to further explore the effects of aging and vaginal delivery on the urethral microenvironment at molecular and single-cell levels on the basis of tissue and cell anatomy.

The urethral sphincter, which is considered the main factor generating urethral pressure, could be divided into the inner smooth muscle and the outer circular striated muscle. The impairment of striated muscle layer is considered the main cause of female SUI on ultrasound examination^22^. Our data showed the structural integrity of striated muscle in the middle-age VDB group was altered leading to impaired urethral closure mechanism. In addition, we also found that estrogen receptor 1 (Esr1) only expressed in the smooth muscle. Previous reports suggest that estrogen replacement therapy is associated with an increased risk of SUI in middle-aged women, but the mechanism is unclear^23,24^. We found that the urethral smooth muscle of middle-age rats expressed abundant ECM proteins such as collagen I, II, IV, et al. In cardiovascular studies, smooth muscle was noted to be converted from contractile to synthetic phenotype under inflammatory or stimulatory conditions^25^. If the same process occurs in the urethra, estrogen may inhibit the structure and function of urethral smooth muscles^26^. Regarding neuromuscular innervation, we found a decreased level of “neuron projection development” genes in urethra muscle with age, and the number of neuromuscular junctions was also decreased.

The urethral wall is rich in loose connective tissue, elastic fiber, collagen and other components. Under the actions of external forces, it can effectively deform and ensure the tightness of the urethral closure^27^. A previous study reported that the content of urethral elastic fibers and collagen in SUI patients was significantly lower than that of normal women^28^. In the current study, we found an increase in the urethra ECM content in middle-age VBD rat suggesting that increase in ECM content may contribute to urethral sphincter insufficiency. Appropriate ECM remodeling, mediated by specific enzymes that are responsible for ECM degradation, ensures a balance between tissue repair and fibrosis^29^. In the urethra, we found that fibroblasts were activated after VDB to form C2 and C3 types of functional cells, corresponding to ECM production and remodeling, respectively. The number of the former increased significantly in the middle-age rat urethra, but the latter did not increase in equal proportion, it may result in an imbalance between ECM production and degradation, leading to fibrosis in the middle-age VBD rat urethra. By building the fibroblast activating lineage, we predict multiple potential regulators of ECM remodeling phenotype fibroblast differentiation, which may be therapeutic targets to improve aging related SUI. In addition, the changes of immune microenvironment cannot be ignored. Our study showed that macrophages increased and were predominantly of M1 type in middle-age rat urethra. M1 macrophages are involved in pro-inflammatory responses, and associated with poor urethral injury repair and fibrosis^30^. In addition, mast cells are an unexpected subcluster that appears in the middle-age rat urethra, mast cell-derived histamine and transforming growth factor beta play an important role in inflammation, fibrosis, and smooth muscle cell dysfunction of many tissues^31,32^. So, regulating the urethral immune microenvironment homeostasis is also another potential direction for the treatment of SUI.

Finally, we found that the aging signature of the urothelium was the most striking across the microenvironment. In young rats 2 weeks after BVD, the transcriptional state has recovered to the level like the sham group. In aged rats, the transcriptional changes in urothelium 4 weeks after VBD were as dramatic as those in fibroblasts and smooth muscles. We postulate that senescence of urothelium which hinders the repair after uerthral injury, may be one of the reasons of incomplete recovery of continence in older female. The urethral epithelium is an important part of the urethral mucosa, which produces mucoid secretions and exerts a valve seat and O-ring like sealing effect in a water faucet with the assistance of submucosal blood vessels^10^. We found that the superficial and intermediate urothelium of the middle-age rats were damaged and shed, leading to the destruction of its barrier structure. However, this phenomenon was not observed in the basal urothelium, which instead expresses a large number of SASP genes, thereby affecting the homeostasis of the whole microenvironment. According to the GO and KEGG enrichment analysis, the senescence of urothelium may be dominant by post-transcriptional regulation, which can be further verified by proteomics and ribosome RNA sequence in the future.

In summary, our research presented an overview of the cellular anatomy of the female urethra, encompassing both young and middle-aged rats, in short term and long-term stress urinary incontinence (SUI) animal models. Analyzing the impact and interactions among different types of urethral cells contributes to a deeper understanding of the underlying mechanisms and establishes foundational data for stress urinary incontinence.

## METHODS

### Experimental Animals and Study Design

Approval for all experiments was granted by the Institutional Animal Care and Use Committee at the University of California, San Francisco. Fourteen female Sprague-Dawley rats, aged 24 and 48 weeks, were procured from Charles River Laboratories (Wilmington, MA, USA). These rats were randomly assigned to two cohorts: (A) Young (24 weeks) and (B) middle-aged (48 weeks). Each cohort was subdivided into 3 groups: sham control (SHAM group), vaginal balloon dilation and ovariectomy (VBDO group), VBDO + BAPN (BAPN group). Each group consists of 7 rats: 6 for functional study and 1 for single cell RNA sequencing. The aging sham control group underwent a sham procedure without vaginal balloon dilation and ovariectomy. The VBDO procedure was performed following established protocols^18^. In brief, under appropriate anesthesia (ketamine/xylazine, 90 mg/kg and 10 mg/kg respectively, intraperitoneally), an 18Fr latex Foley catheter's balloon was inserted into the raťs vagina and filled with 4 ml of water. A 130-g weight was placed at the suspended end of the catheter, creating a consistent force directed toward the pelvic floor. The balloon was retained for 4 hours. After a week, the rats were anesthetized, and bilateral ovaries were surgically removed. Starting from the second week following the initial VBDO, rats in the BAPN groups received intraperitoneal injections of 300 mg/kg BAPN twice a week for 4 weeks. Subsequent to a 1-week washout period, all rats underwent measurement of leak point pressure (LPP). Upon completion of LPP measurement, the rats were euthanized, and their urethras were collected for single-cell RNA sequencing and histological analysis.

### Leak Point Pressure Measurement

Leak point pressure (LPP) was measured using established methods ^33^. In brief, rats were anesthetized with urethane (i.p), and a polyethylene-90 tube was introduced into the bladder dome and secured using a purse string suture. The bladder was gradually filled with heated phosphate-buffered saline (PBS) while recording the volume. The bladder's capacity was recorded at the point of urine leakage. This process was repeated thrice, and the mean bladder capacity was calculated. The bladder was then emptied through aspiration and manual pressure. Changes in intravesical pressure were captured using LabView 6.0 software (National Instruments, Austin, TX, USA) at a sampling rate of 10 samples/sec. The bladder was filled to 40% of its capacity, and incremental manual extravesical pressure was applied until leakage occurred. This process was repeated six times, and the LPP values were documented. Subsequently, the rats were humanely euthanized, and their entire urethras were collected for further analysis.

### Immunofluorescence Staining

Tissue samples were initially fixed using a cold solution containing 2% formaldehyde and 0.002% saturated picric acid in a 0.1 M phosphate buffer at a pH of 8.0. This fixation process lasted for 4 hours, followed by immersion in a buffer solution containing 30% sucrose overnight. Subsequently, the specimens were embedded in OCT Compound (Sakura Finetek USA, Torrance, CA) and stored at a temperature of −70°C. To prepare the fixed frozen tissue specimens for analysis, sections of 10 microns were cut and carefully placed onto SuperFrost-Plus charged slides (Fisher Scientific, Pittsburgh, PA), where they were allowed to air dry for a duration of 5 minutes. These prepared slides were then treated with a mixture of 0.3% H2O2 in methanol for 10 minutes, followed by two consecutive 5-minute washes with PBS. Next, the slides were subjected to a 30-minute incubation at room temperature with a solution containing 3% horse serum in PBS and 0.3% Triton X-100. After the excess solution was removed from the tissue section, the slides were left to incubate overnight at a temperature of 4°C with primary antibodies, including anti-myosin skeletal heavy chain (MHC; 1:500; Mouse, [MY-32] (ab51263), Abcam), anti-smooth muscle actin (SMA; 1:1000; Mouse, Abcam), and anti-Uroplakin-III (UP-III; 1:500; mouse, Santa Cruz Biotechnology, Inc.).For the subsequent steps, secondary antibodies tagged with Alexa-488 and Alexa-594 (1:500; Invitrogen) were used. Nuclei were stained using DAPI, followed by phalloidin (1:500; Invitrogen). The resulting stained tissues underwent examination using fluorescence microscopy.

### Masson's Trichrome Staining, Neuromuscular Junctions, and Phalloidin Staining

To perform Masson's trichrome staining, sections of urethral tissue were initially placed in warm Bouin solution (58°C) for a duration of 20 minutes. Following this, the sections were rinsed and subjected to Weigert Hematoxylin staining for 10 minutes, ensuring that only the nuclei remained stained after thorough rinsing. The subsequent steps included staining with Biebrich Scarlet-Acid Fuchsin for 3 minutes, followed by immersion in phosphomolybdic acid for 45 minutes. Aniline Blue staining was then performed for 3 minutes, succeeded by a 2-minute distilled water rinse and a 2-minute immersion in 1% acetic acid. This was followed by two rounds of a 2-minute distilled water rinse. The sections were then dehydrated through a series of increasing ethanol concentrations, air-dried, and eventually mounted. Neuromuscular junctions were stained using α-bungarotoxin (1:500, Invitrogen), followed by phalloidin. For image analysis, five random fields per tissue section were photographed and documented using a Retiga digital camera along with ACT-1 software (Nikon Instruments Inc., Melville, NY, USA).

### Assay for Senescence-Associated β-Galactosidase Activity

To perform SA-β-Gal staining on cryosections of the urethral tissue, the samples were embedded in OCT, then sectioned at a thickness of 10 µm and allowed to air-dry. After rehydration in PBS, the staining procedure was carried out utilizing the Cell Signaling kit (#9860). This involved an initial fixation period of 12 minutes, followed by incubation in the staining solution at 37°C for a duration of 12 hours.

### Single-cell RNA-seq library preparation and sequencing

Single-cell RNA-seq libraries were prepared using the Chromium Single Cell 3′ Library & Gel Bead Kit v3 (PN-1000094, 10× Genomics) according to the manufacturer’s instructions. Final libraries were sequenced on an Illumina NovaSeq 6000. The raw sequencing reads were processed by Cell Ranger (v.3.1.0) with the default parameters. The reference genome version was Rattus norvegicus mRatBN7.2.106.

### Single-cell RNA-seq library preparation and sequencing Quality control and sample integration

The gene–cell matrix of each sample was used to create a Seurat object with the Seurat package in R. Cells were further filtered according to the following threshold parameters: the total number of expressed genes range from 500 to 9,000; total UMI count range from 0 to 25,000; and proportion of mitochondrial genes expressed, < 20%. Batch correction was performed using the *IntegrateData* function in the Seurat package according to the package manual (https://satijalab.org/seurat/v3.1/pbmc3k_tutorial.html).

### Cell identification and clustering analysis

The merged Seurat objects were scaled and analysed by principal component analysis (PCA). The first 20 PCs were also used to get clustering and perform t-distributed stochastic neighbour embedding (tSNE) dimensionality reduction. The *FindClusters* function in Seurat package with the resolution parameter set as 0.5 was used to cluster the cells. For further analysis of each cluster, we isolated them and performed the above two steps again to get subcluster information.

### Differentially expressed gene calculation and gene enrichment analysis

The Seurat function *FindAllMarkers* (test.use = wilcox; min.pct = 0.1; logfc.threshold = 0.25) was used to identify differentially expressed genes (DEGs) based on the normalised UMI count. Unless otherwise noted, the DEGs in each selected subcluster were calculated based on comparison between that subcluster and the rest of the dataset. GO analysis was performed using the *WebGestalt* website (http://www.webgestalt.org), the Over-Representation Analysis (ORA) or Gene Set Enrichment Analysis (GSEA) was chosen as Method of Interest, and only Biological Process was chosen in Functional Database. The pathway analysis was performed using Ingenuity Pathway Analysis (IPA) software based on the log_2_ (FC) and *P*-values of the DEGs.

### Ligand–receptor interaction and transcription factor network construction

The CellChat package was used for ligand–receptor interaction analysis. The cell–gene matrix was divided according to the six major clusters or five major combined with the subclusters of the other major cluster. The “secreted signalling”, “ECM–receptor” and “cell–cell contact” paired datasets were chosen to analyse the cell communication.

For the transcription factor (TF) regulation network analysis, a total of 1,479 Rat TFs in AnimalTFDB (http://bioinfo.life.hust.edu.cn/AnimalTFDB/) and a cell–gene matrix was taken as input for the GENIE3 package. In the output regulator–target table, only pairs with weights greater than 0.1 were retained and the first 8 TFs were displayed further.

### Statistics and Reproducibility

Statistical significance was calculated by GraphPad Prism 8 software with Tukey's multiple comparisons test of ANOVA. The confidence interval was 95%. Results were considered significant at *P* < 0.05. Statistical analysis between one group and the rest of the scRNA-seq data were made by Wilcoxon (Mann-Whitney) rank sum test; two-tailed, with the Seurat package in R, and the confidence interval is 95%. Statistical parameters are reported in the respective figures and figure legends.

## Supplementary Material

Supplement 1
