# Supplementary material for: Age-Related Changes in Urethral Structure and Responds to Injury: Single-Cell Atlas of a Rat Model of Vaginal Birth Injury induced Stress Urinary Incontinence
Source: Res Sq. 2024 Feb 12:rs.3.rs-3901406. Preprint. [Version 1] doi: 10.21203/rs.3.rs-3901406/v1 (PMC10896383; doi:10.21203/rs.3.rs-3901406/v1)
Supplement: Supplement 1 [file NIHPPRS3901406v1-supplement-1.pdf]

Supplementary Files

This is a list of supplementary files associated with this preprint. Click to download.

- SupplementaryTable1DEGsof8majorclusters.csv
- Supplementary.docx
- nrreportingsummary17074438201.pdf
